# Comparison of drone and vessel-based collection of microbiological water samples in marine environments

**DOI:** 10.1007/s10661-022-10095-8

**Published:** 2022-05-20

**Authors:** Ryan A. Horricks, Cody Bannister, Leah M. Lewis-McCrea, James Hicks, Kiersten Watson, Gregor K. Reid

**Affiliations:** 1 Centre for Marine Applied Research, Dartmouth, NS B2Y 4T5 Canada; 2 Spiri Robotics Inc, Halifax, NS B3J 3N2 Canada

**Keywords:** Uncrewed aerial vehicle, UAV, Drone, Water monitoring, Faecal coliforms

## Abstract

Many water quality metrics cannot be measured in situ and require collection of a physical sample for laboratory analysis. This includes microbiological samples for detection of fecal coliform bacteria in marine and freshwater systems which are a critical component of food safety programs for human consumption of bivalve shellfish worldwide. Water sample collection programs are typically vessel-based which can be time and resource intensive. In Canada, the Canadian Shellfish Sanitation Program aims to avoid consumption of contaminated molluscan bivalves by monitoring fecal coliform bacteria through vessel-based water sample collection. Uncrewed aerial vehicles or drones are becoming more commonly used for water sample collection given their relatively low cost but are rarely used to support microbiological analyses. A prerequisite for the acceptance of a new collection method for a regulatory program is to determine if the method of sample collection affects results. To assess this potential, we designed, developed, and tested a sampling device attached to the underside of a drone to collect water samples for bacteriological analysis. Drone and vessel-based samples were collected in the same location, at the same 20-cm depth, within a minute apart, at ten different geographic locations in coastal Nova Scotia waters to compare fecal coliform counts. Bacterial count estimates obtained from drone-collected samples were not significantly different than estimates obtained from vessel-collected samples (*p* < 0.5). Results from this study suggest novel water sampling techniques using drones could supplement or replace traditional vessel-based sampling methods.

## Introduction

There are numerous water quality metrics that cannot be measured in situ or through remote sensing. In these instances, a physical sample must be collected and subsequently transported to a laboratory for analyses. Identification and quantification of most microbiological organisms, such as fecal coliform bacteria in marine systems, fall into this category. Sampling for fecal coliform is a cornerstone of food safety programs for bivalve shellfish destined for human consumption worldwide (e.g. Kucuksezgin et al., 2010; Munoz et al., 2010; Rubini et al., 2018; Shin et al., 2019; Umesha et al., 2008). Coastal marine shellfish aquaculture operators and wild harvesters are reliant on these sampling programs to sell their product. The potential for industry growth can, therefore, be limited by program sampling capacity. Mechanisms which can increase the capacity of water sample collection may ultimately be needed for industry expansion.

Uncrewed aerial vehicles or drones have been successfully used for water sample collection to increase sampling efficiency and improve safety at reduced cost compared to traditional sampling methods (Ore et al., 2015; Song et al., 2017; Terada et al., 2018). These systems are lightweight, cost-effective, and highly customizable (Wu et al., 2019). While several drone water-sampling initiatives have sampled physiochemical water quality (e.g. Koparan et al., 2018a, b; Song et al., 2017), only recently have the collection of aquatic microorganisms such as bacteria (Benson et al., 2019) and harmful algae (Kimura et al., 2019) been successfully collected for analysis. There are currently several research initiatives underway investigating drone capacity to sample water quality (e.g. Balpataky, 2018; DroPLEtS, 2020); however, none is presently investigating the potential of drones to meet food safety sampling requirements in Canada.

**Fig. 1:**
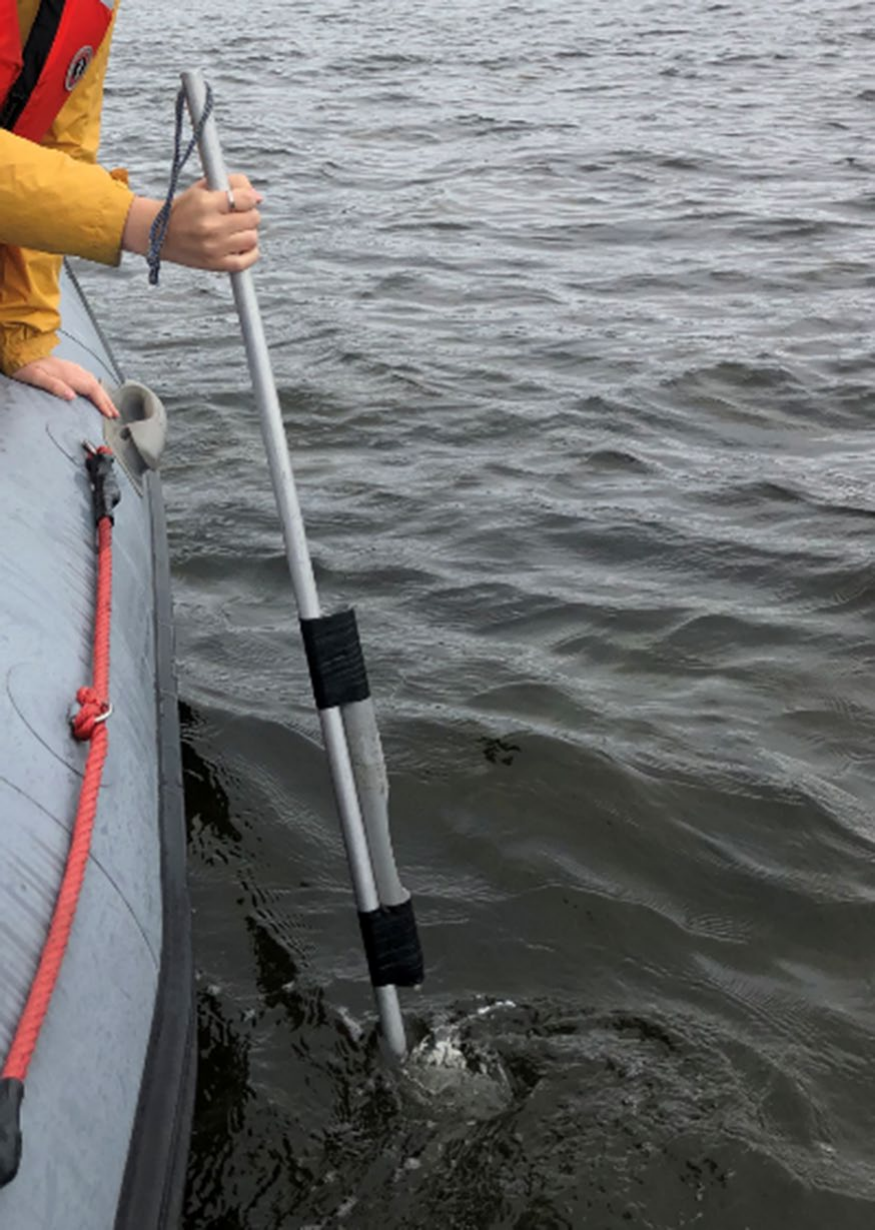
Vessel-based water collection using an aluminum rod with a gauge to collect samples at a depth of 20 cm

The Canadian Shellfish Sanitation Program (CSSP) is a federal food safety program jointly administered by the Canadian Food Inspection Agency (CFIA), Environment and Climate Change Canada (ECCC), and Fisheries and Oceans Canada (DFO). The CSSP aims to minimize health risks associated with the consumption of contaminated bivalve molluscan shellfish such as mussels, oysters, and clams by monitoring bacteriological water quality (e.g. *Escherichia coli* contamination), identifying and evaluating pollution sources, and classifying shellfish harvesting areas (CFIA, 2019). Currently, ECCC collects water samples for the CSSP from a small watercraft using a Nalgene® bottle attached to the end of an aluminum rod (Fig. 1) to estimate bacterial concentration using the fecal coliform most probable number (MPN) method (APHA, 2017; CFIA, 2020). Currently, ECCC has reached its sampling capacity and cannot classify additional areas as safe for harvest, thereby limiting the potential growth of shellfish aquaculture and wild-harvesting areas in Canada.

In 2019, the Atlantic province of Nova Scotia had more than 165 commercial bivalve aquaculture leases distributed throughout the province, producing 1,830 metric tonnes of product with a farm gate value of over $5.3 million (Nova Scotia Department of Fisheries & Aquaculture, 2020). Shellfish production in Nova Scotia has room to grow; however, the capacity to classify new shellfish harvesting areas under the CSSP is a major limiter of industry expansion. Given the limitations with ECCC’s current water quality sampling methodology, we designed, developed, and tested a water sampling device for a drone to enable more cost-effective and rapid water sample collection to meet the same quality standards as traditional vessel-based sampling. The objective of the present study was to compare results from traditional vessel-based water sampling with novel drone-based water sampling to determine if drones are an acceptable alternative for sample collection under the CSSP.

## Methods

### Study sites

Six study sites in pre-existing Nova Scotian CSSP sampling areas and four sites in previously unsampled areas were selected for comparison between traditional vessel and novel drone sampling methods (Table 1). All study sites in the pre-existing Nova Scotia CSSP areas are currently monitored by the CSSP and have been monitored since the 1980s (Environment and Climate Change Canada, 2018).

**Table 1:** Study site locations, site inclusion in the Canadian Shellfish Sanitation Program, sample collection dates, and number of replicates collected using each method in Nova Scotia, Canada

Site name	CSSP	Latitude	Longitude	Sampling date	Replicates
Annapolis Basin	Y	44.7510	-65.5130	2020–11-18	3
Musquodoboit River	Y	44.7911	-63.1358	2020–11-19	3
Musquodoboit Harbour	Y	44.7911	-63.1358	2020–11-19	3
Fox Point	Y	44.6151	-64.0568	2021–02-04	5
St. Margarets Bay	Y	44.6370	-64.0581	2021–02-04	3
Sober Island	Y	44.8464	-62.4642	2021–02-10	7
Shelburne Harbour 1a	N	43.7585	-65.3242	2021–03-10	5
Shelburne Harbour 2a	N	43.7572	-65.3238	2021–03-10	3
Shelburne Harbour 1b	N	43.7585	-65.3242	2021–06-23	5
Shelburne Harbour 2b	N	43.7578	-65.3233	2021–06-23	5

### Platform specifications and sampler design

The drone used in this study was the Spiri Mu (Spiri Robotics Inc., Halifax, Nova Scotia), a quad rotor drone that runs Ubuntu Linux and ROS and is optimally configured for autonomous missions and deep learning. Basic navigation is supported by a flight control module (PixRacer, Mayan Robotics LLC, California, USA), with a barometer and several redundant inertial measurement sensors (accelerometer, gyroscope, magnetometer) connected to a global navigation satellite system unit, a downward-facing time-of-flight sensor, and an optional ultrasonic sensor. The Spiri Mu is equipped with a companion computer housing a TX2 (Nvidia, California, USA) system-on-chip connected to its forward stereoscopic cameras. This companion computer supports machine vision and learning capabilities, as well as autonomous navigation, mission planning, obstacle avoidance, and communications with other autonomous systems and remote servers. The 17-cm diameter drone has a nominal takeoff weight of 1.5 kg and a maximum weight of 2.5 kg with a thrust to weight ratio of 3.2:1 and an average flight time of 24 min. The Spiri Mu can operate in winds up to 15 m s^−1^, gusting to 20 m s^−1^, and in ambient temperatures from − 10 to 40 °C. The drone is piloted using a flight controller (Mayan Robotics, California, USA) and uses a global navigation satellite system with a maximum Wi-Fi range of 200 m to the nearest router. When outside of Wi-Fi range, it operates on radio frequency at a 900 MHz bandwidth and 4G/LTE within 30 km of the nearest cell tower. The system is powered by four Li-ion cells in series, at a voltage ranging from 16.8 V when fully charged to 12.0 V when operationally depleted. Depleted batteries are typically exchanged manually for fresh ones between flights and recharged.

**Fig. 2:**
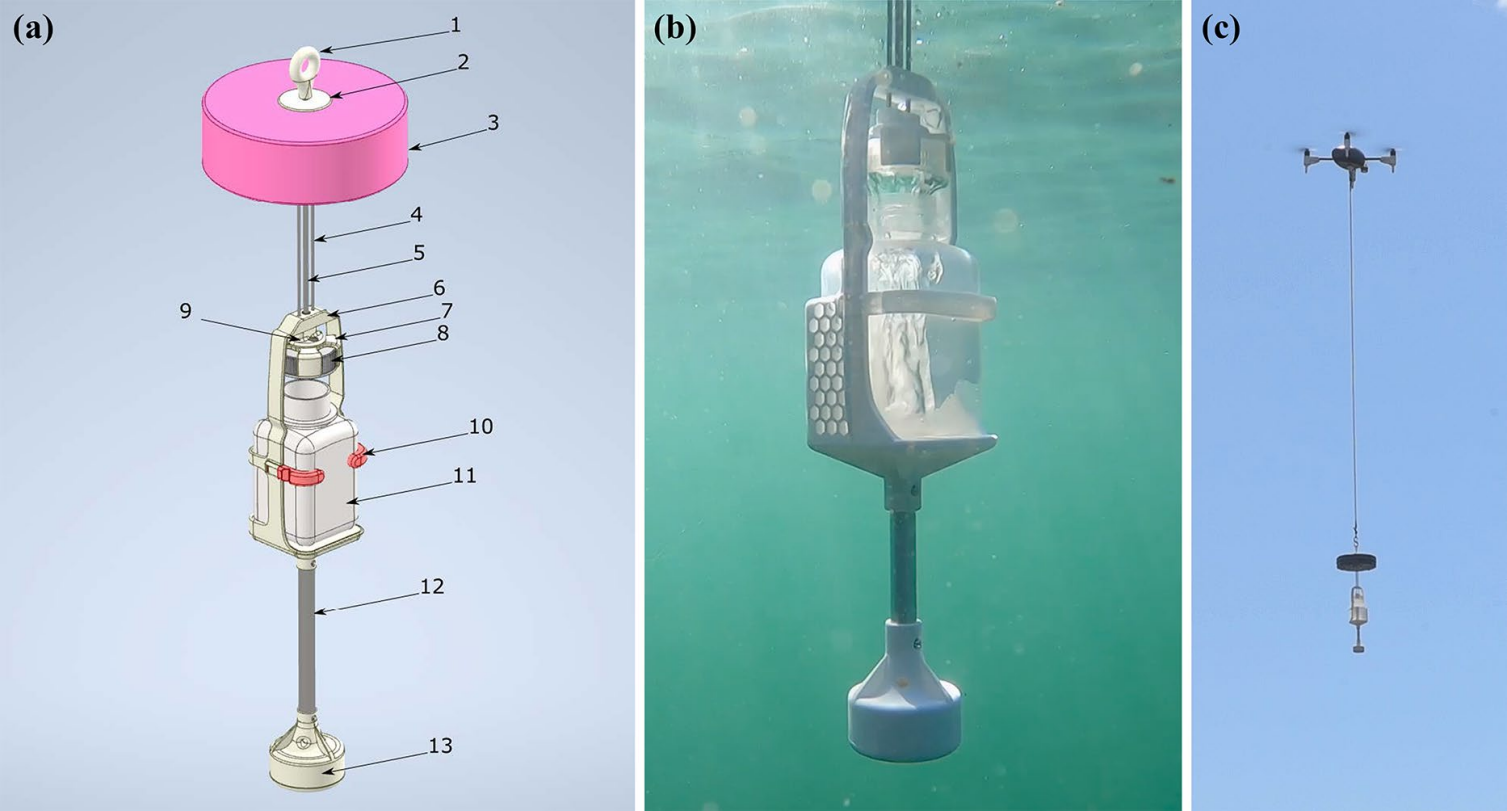
Water sampling device developed in partnership with Spiri Robotics Inc. **A** Three-dimensional rendering of the water sampling device showing the following: 1—eyebolt, 2—buoy cap, 3—buoy, 4—linear rails, 5—support rod, 6—bottle cage, 7—cap holder, 8—bottle cap, 9—cap connector, 10—retention arms, 11—sample bottle, 12—weight rod, 13—weight capsule. **B** Underwater photo of water sampling device collecting a water sample. **C** Water sampling device attached to a Spiri Mu

The Spiri Mu (Spiri Robotics Inc.) was equipped with a custom-built water sampling attachment (Fig. 2) that weighed 500 g when empty. The attachment was designed to collect a water sample at a depth of 20 cm without the use of electronic actuation. To operate in the marine environment, the device was designed to be robust and able to withstand extreme weather conditions, including large waves, high winds, and strong currents. A linear rail system and 18-cm float were used to actuate the lid and open it at a pre-determined depth of 20 cm to comply with current CSSP sampling standards (CFIA, 2019; ECCC, 2020).

### Flight planning and method of operation

A drone operator can interface with the Spiri Mu (Spiri Robotics Inc.) via a remote controller, cell phone, tablet, computer, or terminal shell. The open-source software, QGroundControl (Dronecode Project, Inc.), was used in the present study. This allowed for piloting through joystick control, semi-autonomous waypoints, and semi-autonomous area coverage via a map-based graphical interface. To avoid potential damage to the drone or water sampler, the Spiri Mu (Spiri Robotics Inc.) used the default settings to return to the launch location at 7% battery capacity and land at 5%. Vertical launch and landing capabilities allowed for efficient sample collection in the field with minimal space requirements.

### Vessel-based sample collection

Vessel-based sample collection was conducted using the method currently approved by the CSSP (CFIA, 2019; ECCC, 2020), which was modified from the method described in Standard Methods for the Examination of Water and Wastewater (APHA, 2017). The modified procedure uses a 250 mL Nalgene® bottle attached to the end of an aluminum rod with a secured cap (Fig. 1). The bottle is rapidly plunged into and removed from the water, the cap is removed, and the bottle is rapidly plunged back into the water without a cap, to a 20-cm depth and held 5 s for filling (CFIA, 2020; ECCC, 2020). The bottle is returned to the surface, capped, and stored on ice for transit to the laboratory.

### Water sampling experimental design

Sample temperature and collection time were recorded for all samples. For the site temperature control, a separate water sample was collected prior to bacterial sampling using the vessel-based method. Sterilized laboratory-grade 250 mL Nalgene® bottles were provided by ECCC’s ISO17025 accredited microbiology laboratory and used for all samples. Prior to launch, the sterilized bottles were manually inserted into the drone sampler and the sample station coordinates were entered in the flight operator software to determine automated flight paths. Drones were programmed on location to launch from land, ascend to the 10 m flight altitude, fly to station GPS coordinates, hover, and descend to lower the sampling device into the water. The drone maintained position with the sampler submerged at a depth of 20 cm for approximately 5 s to allow the bottle to fill before lifting the sampler from the water, ascend to flight altitude, and return to the launch location. As soon as the drone safely vacated the sampling station, the vessel approached the same station and collected a sample using the standard vessel-based CSSP methodology (CFIA, 2019; ECCC, 2020) within 1 min of drone sample collection. At the launch location, the drone stopped, descended to, and hovered at 1.5 m to enable manual removal of the Nalgene® bottle from the water sampler. The bottle cap was tightened, and the bottle stored on ice for transit to the laboratory. A minimum of three replicates was initially collected using each method, but replicate number was increased over the course of the study to accommodate increased variation encountered with high MPN samples (Table 1). The temperature of the control sample was recorded at the laboratory to ensure that water temperature had not changed by > 10 °C in transit (CFIA, 2020). MPN bacterial concentration was estimated for all samples using the multiple tube fermentation technique for members of the coliform group method #9221 (APHA, 2017).

### Statistical analysis

A one-way ANOVA was used to compare mean bacterial estimates between the two sampling methods. All statistical analyses were conducted using R Version 4.1.0 (R Core Team, 2021) with a significance level of *α* = 0.05.

## Results

**Fig. 3:**
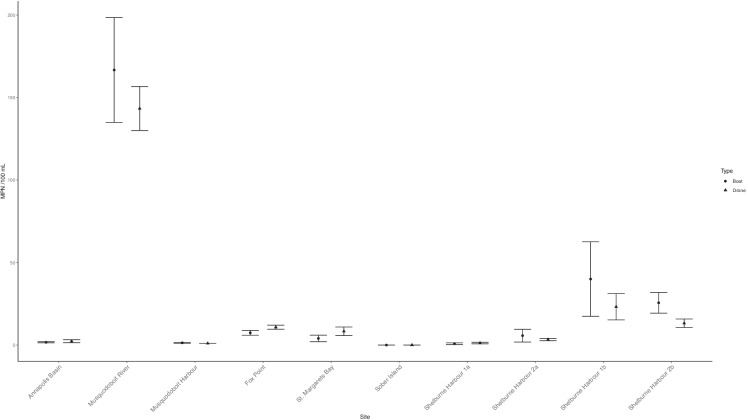
Mean estimated bacterial concentration (most probable number) ± SE at water sample collection locations in Nova Scotia, Canada, showing no significant differences between groups (*F*_(1, 82)_ = 0.236, *p* = 0.628)

Mean return flight distance per site was 332.0 m ± 28.0 and return flight time ranged from 1:12 to 23:55 min with a mean return flight time of 4:24 min. Air temperatures ranged from − 10 to 21 ℃ with a mean temperature of 1.3 ± 1.4. There were no significant differences in estimates of bacterial concentration between samples collected using the vessel and drone methods (*F*_(1, 82)_ = 0.236, *p* = 0.628). A post hoc power analysis showed that a total of 84 samples in two equal sized groups of *n* = 42 achieved a power of 0.876 at a significance level of *α* = 0.05. Mean MPN of bacteria collected from vessel samples was 21.6/100 mL ± 7.34 (*µ* ± *SE*) while mean MPN of bacteria collected from drone samples was 17.1/100 mL ± 5.72 (*µ* ± *SE*) (Fig. 3).

## Discussion

### Drone vs. vessel sampling

The drone successfully collected samples that were not significantly different from vessel-collected samples. While efforts were made to collect samples from areas with suspected high MPN estimates, many locations had low MPN estimates. Some locations with low MPN estimates may have been a function of cold winter water temperatures during collection (Table 1), when bacterial estimates would be expected to be low. Sample collection for this project also occurred during the COVID-19 pandemic, which significantly impacted travel and field personnel, limiting opportunities to source locations and seasons more likely to reflect elevated MPN in samples. Collecting samples in areas with historically high MPN estimates each month over the course of a year would be ideal; however, this was beyond the financial and temporal scope of this proof-of-concept project.

Statistical power can be problematic for sample means with high coefficients of variation, which is common for biological samples such as those in this study (Fig. 3). At the three contaminated locations in this study (MPN > 14/100 mL), the coefficient of variation (CV) for vessel-based sample means within each location ranged from 33 to 126%. Even at the lower CV of 33%, in order to detect a difference greater than one standard deviation between sample means, a minimum of approximately 20 replicates would be required to achieve a satisfactory statistical power of 0.80 for a two-tailed test (Berndtson, 1991). This is largely impractical for single location comparisons and may explain why regulatory compliance for CSSP applies median values to samples collected across time (CFIA, 2020), presumably to circumvent issues with high variability and skewed distributions. To achieve a safe for harvest classification, the CSSP requires the median MPN of 15 samples be < 14/100 mL and not more than 10% of the samples may have MPN estimates > 43/100 mL when conducting a comprehensive review of an area (CFIA, 2020). A previously closed area may be re-opened if three consecutive acceptable samples are collected over a 14-day period and show a downward trend in bacterial MPN estimates (CFIA, 2020).

### Methodological differences

There are four methodological differences between the techniques that should be noted. These are (1) the sample bottle rinsing procedure; (2) capped or uncapped sample bottle submersion; (3) the time between sample collection and cap tightening; and (4) the method used for creating headspace in the sample container.

The vessel-based sampling protocol requires an immersion ‘rinse’ of the capped sample bottle prior to cap removal and water sample collection (ECCC, 2020). With the drone method, there was not a separate ‘rinse’ procedure, instead, the bottle was immersed for 20 s and, upon withdrawal, an actuator rested the lid back on the mouth of the bottle, which was not screwed on tightly until the drone returned to the operator. There is nothing to suggest that the slight difference in rinsing procedure would not provide similar safeguards against accidental contamination.

Another difference is that the cap on the drone-collected sample is not tightly sealed until it has returned to shore. The difference in cap threading time is expected to be less than two minutes in most instances. While it is theoretically possible that airborne contaminants could enter between the convex cap and bottle mouth during transit time, fecal coliform is not expected to be airborne. The exception would be a chance encounter with bird excrement for which there would be visible evidence of contamination, necessitating collection of a new sample.

The methods also differ as the vessel-based method submerges the sample bottle without a cap while the drone-based method keeps the cap pressed against the mouth of the bottle until the 20-cm sampling depth is reached. It is assumed that some surface water could mix in with the vessel-based procedure but far less so than with the drone procedure. To further reduce the risk of contamination on the external surface of the bottle using either method, personnel could wear sterile gloves when interacting with the sample bottles.

Finally, samples collected by field personnel from a vessel using a hand-held aluminum rod are submerged until each sample has been filled with 200–250 mL of seawater (ECCC, 2020). The amount of time required to do this without overfilling the bottle is subjective, which can lead to over-filling the sample bottle. In the event too much water is collected, field personnel are instructed to pour off the excess in the field to provide appropriate headspace in the bottle that will allow for agitation of the sample at the laboratory (CFIA, 2019; ECCC, 2020). It was not possible to do this at the sampling station for the drone-collected samples. Instead, over-filled bottles can easily be decanted at the landing site to ensure appropriate head space.

### Advantages of drones for water sample collection

In addition to reduced costs and improved sample collection safety compared to vessel-based collection, drones are now technologically developed enough to use machine learning in real-time, capable of traveling in a ‘flock’, and enable modular adaptation for payload change reflective of the sampling environment. Machine learning can be applied for on-the-fly decision-making. For example, significant amounts of algae in the water sample may yield higher results of fecal coliforms as the bacteria may attach to algal cells and artificially inflate MPN results (ECCC, 2020). Flocking capabilities increase collection efficiency lending to larger sample numbers with the ability to delineate contaminated areas. Drone platforms could be equipped with additional sensors, such as chlorophyll sensors, that would communicate with the drone, causing it to adjust the mission if a threshold chlorophyll concentration was exceeded. Increases in advanced autonomy and decision-making at the sample collection location will allow for the potential to collect other environmental data such as salinity, temperature, and/or conductivity which may affect real-time decision-making. Drone processors have already developed to the point that they are able to complete automated landing procedures and sample collection in unique environmental parameters (James Hicks, Spiri Robotics Inc., *pers. comm.*, 2021). This processing power could eventually contribute towards a single operator dispatching multiple drones traveling in a ‘flock’ to collect simultaneous samples for more efficient sampling of novel areas.

Due to the modular build of the Spiri Mu, it was straightforward and affordable to adapt many of its components to unique environments. For the requirements of the present study, the electronics and selected body components were adapted to mission-specific parameters and conditions such as salinity and humidity. These adjustments could be easily adapted to a diverse set of environmental conditions in a variety of marine and freshwater environments. The design provided a field-adaptable platform to refine technology and thresholds which accommodated mission-specific requirements, conditions, and processes.

### Application of drones for microbiological sampling

At the time the present study was conducted, ECCC had implemented a pilot program allowing shellfish growers to collect and submit samples to a certified laboratory at their own expense for area classification (CFIA, 2019). Vessel sampling at an existing shellfish farm can be straightforward as staff and vessels are typically available on site, with sample transit and laboratory analysis the predominant expenses. However, vessel sampling collection at novel locations can become logistically and resource expensive, mainly due vessel travel time required to collect a minimum of fifteen samples in a proposed novel area every three to four weeks between March and November over a two-year period (CFIA, 2019). The potentially high cost of vessel-based sampling could be mitigated through the adoption of sampling drones that can be easily directed from shore by a single user. Shellfish harvesting areas that are closed because classification is unknown due to a lack of sampling capacity could be sampled more frequently, to determine if they can be classified for harvest. Study results suggest that the minor differences between vessel and drone collection methods may result in negligible outcomes for shellfish harvest classification. However, the use of drones to collect water samples for the CSSP is not currently approved by ECCC. Additional consultation and testing may be warranted prior to regulatory approval of this novel technique.

## Conclusions

Bacterial count estimates obtained from drone-collected samples were not significantly different than estimates obtained from vessel-collected samples. Results from this study suggest novel water sampling techniques using drones could supplement or replace traditional vessel-based sampling methods.

## Data Availability

Data will be made available on reasonable request.
